# Mutant p53 Cooperates with Knockdown of Endogenous Wild-Type p53 to Disrupt Tubulogenesis in Madin-Darby Canine Kidney Cells 

**DOI:** 10.1371/journal.pone.0085624

**Published:** 2013-12-27

**Authors:** Yanhong Zhang, Wensheng Yan, Xinbin Chen

**Affiliations:** Comparative Oncology Laboratory, Schools of Medicine and Veterinary Medicine, University of California Davis, Davis, California, United States of America; Vanderbilt University Medical Center, United States of America

## Abstract

Mutation of the p53 gene is the most common genetic alteration in human malignances and associated clinically with tumor progression and metastasis. To determine the effect of mutant p53 on epithelial differentiation, we developed three-dimensional culture (3-D) of Madin-Darby canine kidney (MDCK) cells. We found that parental MDCK cells undergo a series of morphological changes and form polarized and growth-arrested cysts with hollow lumen, which resembles branching tubules *in vitro*. We also found that upon knockdown of endogenous wild-type p53 (p53-KD), MDCK cells still form normal cysts in 3-D culture, indicating that p53-KD alone is not sufficient to disrupt cysts formation. However, we found that ectopic expression of mutant R163H (human equivalent R175H) or R261H (human equivalent R273H) in MDCK cells leads to disruption of cyst polarity and formation of invasive aggregates, which is further compounded by knockdown of endogenous wild-type p53. Consistently, we found that expression of E-cadherin, β-catenin, and epithelial-to-mesenchymal transition (EMT) transcription factors (Snail-1, Slug and Twist) is altered by mutant p53, which is also compounded by knockdown of wild-type p53. Moreover, the expression level of c-Met, the hepatocyte growth factor receptor and a key regulator of kidney cell tubulogenesis, is enhanced by combined knockdown of endogenous wild-type p53 and ectopic expression of mutant R163H or R261H but not by each individually. Together, our data suggest that upon inactivating mutation of the p53 gene, mutant p53 acquires its gain of function by altering morphogenesis and promoting cell migration and invasion in part by upregulating EMT and c-Met.

## Introduction

 Wild-type p53, a tumor suppressor, plays an important role in cell-fate determination through the regulation of the cell cycle, programmed cell death, differentiation or senescence [1,2]. Thus, inactivation of p53 leads to many alterations, including cell transformation [3,4] and developmental abnormalities [5,6]. Wild-type p53 can be inactivated by a number of mechanisms including deletion, translocation, point mutation and through interaction with viral and cellular oncoproteins [7-10]. Among these, mutation of the p53 gene is the most common genetic alteration in a wide spectrum of human malignancies [11-13]. The majority (75%) of p53 mutations is missense mutations, many of which are clustered at the DNA-binding domain [14]. Six p53 mutations are described as “hot-spot” because they are the most frequent mutations in human cancers [15]. These mutants are defective in binding to consensus wild-type p53 responsive element and also defective in transactivation of wild-type p53 target genes [16]. Based on the effect on the core DNA-binding activity, these mutations can be classified into two main groups, conformational mutation and contact-site mutation. The conformational mutants, such as R175H and R249S, have an altered conformation of the core DNA-binding domain [17]. The contact-site mutants, such as R273H and R248W, have mutations at residues that directly contact target DNA [18].

 Mutant p53 can form a heterotetramer with wild-type p53 and inhibit wild-type p53 to act as a tumor suppressor [19,20]. In addition, increasing evidence demonstrates that p53 mutants gain new transforming abilities and promote tumorigenesis independent of wild-type p53 [21]. For example, mutant p53 is capable of promoting tumor cell proliferation [22], enhancing chemo-resistance [23] and inducing gene amplification [24]. Additionally, cells carrying a germline p53 mutation are prone to reprogramming and exhibit properties of cancer cells instead of normal stem cells [25]. Moreover, knock-in mice harboring mutant R175H or R273H are prone to metastatic tumors [26]. Recently, we and others showed that mutant p53 is found to acquire gain of function activities via induction of epithelial-to-mesenchymal transition (EMT) [3,5,27-29].

To determine mutant p53 gain of function in morphogenesis and tumorigenesis, we examined mutant p53 activity using 3-D culture model of Madin-Darby canine kidney (MDCK) cells. MDCK cells in 3-D culture undergo a series of morphological changes and form polarized and growth-arrested cyst structures with hollow lumen, which re-differentiates into normal tubules upon induction by hepatocyte growth factor (HGF) [30,31]. Here, we found that ectopic expression of mutant R163H or R261H disrupts normal tubular architectures, which is enhanced by knockdown of endogenous wild-type p53. We also found that mutant p53 induces EMT and c-Met expression. Collectively, our results suggest that upon inactivating mutation of the p53 gene, mutant p53 disrupts normal cell morphogenesis at least in part via induction of EMT and c-Met.

## Materials and Methods

### Reagents

Bovine collagen solution (3.2 mg/mL) was purchased from Advanced Biomatrix (Poway, CA). MEM medium and non-essential amino acid were purchased from Invitrogen (Carlsbad, CA). Recombinant human hepatocyte growth factor (HGF) was purchased from Sigma (St. Louis, MO). 

### Plasmid Construction and Cell Line Generation

To generate vectors expression a shRNA against canine p53 under the control of the U6 promoter, two 62-base oligos were annealed and then cloned into pBabe-U6 shRNA expression vector. The resulting plasmids were designed as pBabe-U6-shp53, which carries a puromycin selection marker [32]. To generate a construct expressing siRNA-resistant mutant p53, an 1,145-bp DNA fragment containing the entire open reading frame of p53, in which four nucleotides (underlined) within siRNA targeting region (5′- GCAATCTACCTCTCGCCAT-3′) was replaced with 5′- GCAATCAACATCACGACAT-3′, was amplified with forward primer P1, 5′- AAGCTTACCATGCAAGAGCCACAGTCAGAG-3′, and reverse primer P2, 5′- CTCGAGCACATCTGTACCATGCAAAGT-3′. The resulting fragment encoding siRNA-resistant p53 was confirmed by sequencing and cloned into pcDNA4, and the resulting plasmid was designated as pcDNA4-siRNA-resistant mutant p53 (Figure S3). Next, this plasmid was used as template to generate pcDNA4 plasmids expressing siRNA-resistant tumor-derived hot-spot p53 mutants (R163H and R261H), in which residue arginine was replaced by histidine. For p53 (R163H), fragment 1 was amplified with forward primer P1, and reverse primer P3, 5′-CATGGTGGGGGCAGTGCCGCACAAC-3′; fragment 2 was amplified with forward primer P4, 5′- GTTGTGCGGCACTGCCCCCACCATG-3′, and reverse primer P2. Then, fragments 1–2 were mixed together as a template and amplified with primers P1 and P2. For p53 (R261H), fragment 1 was amplified with forward primer P1, and reverse primer P5, 5′- GGCACAAACGTGTACCTCAAAGCTG-3′; fragment 2 was amplified with forward primer P6, 5′-TTGAGGTACACGTTTGTGCCTGTCC-3′, and reverse primer P2. Then, fragments 1–2 were mixed together as a template and amplified with primers P1 and P2. The resulting fragments encoding p53 (R163H) and p53 (R261H) were confirmed by sequencing and cloned into pcDNA4. To generated mutant p53-producing cell lines, pcDNA4-mutant p53 was transfected into MDCK cells. The resulting mutant p53-producing cell lines were selected with Zeocin and confirmed by Western blot analysis. To generate stable p53-KD cell lines with mutant p53 overexpression, pBabe-U6-siRNA was co-transfected with pcDNA4-mutant p53 into MDCK cells. The resulting cell lines were selected with puromycin and Zeocin. Both p53-KD and mutant p53 expression were confirmed by Western blot analysis and RT-PCR assay. 

### Cell culture

The MDCK cell line was obtained from American Type Culture Collection (ATCC, Manassas, VA) and cultured in MEM medium supplemented with 10% fetal bovine serum and 1% non-essential amino acid. The overlay 3-D culture was carried out as described previously with some modifications [33]. Briefly, 12-well culture plates were pre-coated evenly with 1.0 mg/mL pre-mixed collagen gel and then incubated at 37 °C for 30 min to allow the collagen gel to solidify. MDCK cells or MDCK cells with p53-KD, p53-R163H, p53-R261H, p53-KD-R163H and p53-KD-R261H (5,000 cells) suspended in 1.0 mL collagen gel mixture were seeded on the top of pre-gelled layer, and then incubated for 30 min at 37 °C to solidify. Complete growth medium was gently added to the top of each gel and incubated at 37 °C in a humidified 5% CO_2_. Culture medium was renewed every third day. For induction of tubulogenesis, culture medium plus 10 ng/mL of HGF was added to the culture plate.

### Western blot analysis

Western blot was performed as described [5]. Antibodies used were purchased from Santa Cruz Biotechnology (anti-p53 (FL393), anti-β-catenin (E-5), anti-Snail-1, anti-Twist, and c-Met, Santa Cruz, CA), Cell Signaling (anti-Slug, Danvers, MA), BD Transduction Laboratories (anti-E-cadherin, San Jose, CA), Sigma (anti-actin, St. Louis, MO), and BioRad (secondary antibodies against rabbit or mouse IgG conjugated with HRP, Life Science Research, Hercules, CA). Experiments were repeated at least three times.

### RT-PCR analysis

Total RNA was extracted from cells using TRIzol (Invitrogen Life Technoloogies, Grand Island, NY). cDNA was synthesized using M-MLV Reverse Transcriptase Kit (Promega Corporation, Madison, WI) according to manufacturer’s protocol. The mRNA level of wild-type p53 was measured by PCR. The special primers to detect wild-type p53 are sense 5′-GTGCCTCACAGAGTGCAAAA-3′, and antisense 5′-CCTGAATGTTGGGAGCATTT-3′. The *Actin* gene was chosen as a loading control and detected with primers 5´-ctgaagtaccccatcgagcacggca-3´ (sense) and 5´-ggatagcacagcctggatagcaacg-3´ (antisense).

### Colony formation assay

MDCK cells were cultured in a 6-well plate for ~12 d and then fixed with methanol/glacial acetic acid (7:1) followed by staining with 0.1% crystal violet. Experiments were conducted in triplicate.

### Wound healing assay

Cells were grown in a 6-well plate for 24 h. The monolayers were wounded by scraping with a P200 micropipette tip and washed two times with PBS. At specified time points after the scraping, cell migration was captured using phase contrast microscopy and cell monolayers were photographed using a Canon EOS 40D digital camera (Canon, Lake Success, NY). Migration rate of cells was measured by averaging the time required to close the borders of cells. Six regions were analyzed in each well, and the result was expressed as the mean ± SD.

### Statistical analysis

Data were presented as Mean ± SD. Statistical significance was determined by Student’s *t* test. Values of P < 0.05 were considered significant.

## Results

### Ectopic expression of conformational mutant p53 R163H disrupts normal cyst formation in 3-D culture

MDCK cell line contains wild-type p53 and possesses the ability to form cyst structures when cultured in 3-D collagen gel [30]. Upon induction with HGF, these cysts develop into branching tubules through partial-EMT, cell proliferation, and re-differentiation, a process that resembles kidney tubulogenesis *in vivo* [30,31]. We showed that when cultured in a 3-D collagen gel, MDCK cells formed a polarized cyst structure, which then formed tubular networks upon stimulation with HGF (Figure S1), which is consistent with our published studies [32]. In addition, we showed that knockdown of endogenous wild-type p53 led to increased cell proliferation and migration in 2D culture, but p53 knockdown alone was insufficient to alter tubulogenesis in 3-D culture (Figure S2), which is also consistent with our published studies [32]. 

**Figure 1:**
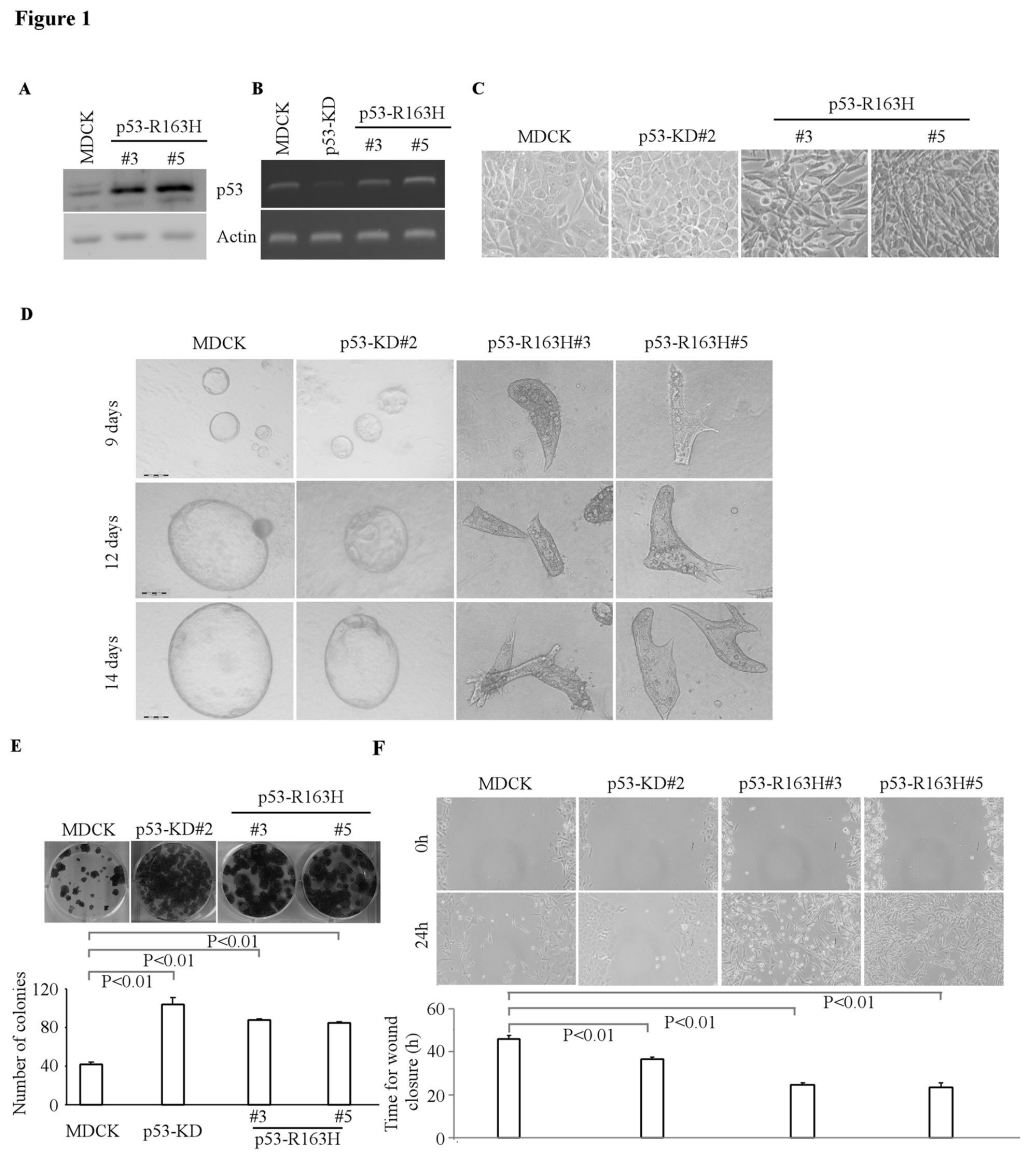
Overexpression of mutant p53 R163H disrupted tubular formation in 3-D culture. **A**, Generation of MDCK cell lines in which siRNA-resistant mutant p53-R163H was stably overexpressed (clones 3 and 5). The level of p53-R163H was determined by Western blotting. **B**, The level of wild-type p53 transcripts was determined by RT-PCR. **C**, Representative images of MDCK cells, MDCK cells with p53 knockdown, or MDCK cells with mutant p53 (R163H) in 2-D culture (200×). **D**, Representative images of MDCK cells, MDCK cells with p53 knockdown, or MDCK cells with mutant p53-R163H in 3-D culture for 6 d or 12 d. Scale bar: 100 µM. **E**, Top panel: colony formation assay was performed with MDCK cells, MDCK cells with p53 knockdown, or MDCK cells with mutant p53-R163H. Bottom panel: the number of colonies was counted and presented as Mean ± SD from three separate experiments. **F**, Wound healing assay was performed with MDCK cells, MDCK cells with p53-KD, or MDCK cells with mutant p53-R163H. Top panel: cell migration was determined by visual assessment of cells migrating into the wound for 24 h using a phase-contrast microscopy. Bottom panel: the time required for wound closure was measured and presented as mean ± S.D. from three separate experiments.

Mutation of p53 is a frequent event in renal cell carcinomas (RCC) and mutant p53 is a prognostic indicator in RCC [34,35]. Consistent with that in human, p53 “hot-spot” mutations were also found in canine TP53 gene, such as R163H (equivalent to R175H in human) and R261H (equivalent to R273H in human) [36]. To examine whether conformational p53 mutant R163H affects cyst formation in MDCK cells, we generated multiple MDCK cell lines in which R163H mutant was ectopically expressed (Figure 1A). To detect the level of wild-type p53 in these cell lines, RT-PCR was performed by using special primers that located in 3’UTR of wild-type p53. We found that the mRNA level of wild-type p53 decreased in MDCK-p53-KD cells, but remain unchanged in MDCK-R163H cell lines compared to that in MDCK cells (Figure 1B). In addition, we found that MDCK cells with R163H mutant exhibited spindle-shaped morphology in 2-D culture, which represents the property of mesenchymal cells (Figure 1C). We also found that in 3-D culture, the frequency of normal cyst formation was decreased and the orientation of cell division became random in mutant R163H-producing MDCK cells (Figure 1D). Additionally, we found that accompanied with the spindle-like cyst structures, R163H-producing MDCK cells exhibited increased cell growth based on clone number and size by colony formation assay (Figure 1E) and cell migration by wound healing assay (Figure 1F). Given that the orientation of cell division is extremely important in influencing the formation and number of lumens within a cyst [37], our data implicated that ectopic expression of mutant R163H disrupts cell polarity in 3-D culture and promotes cell growth and migration in 2-D culture. 

### Ectopic Expression of Contact-Site Mutant p53 R261H Disrupts Normal Cyst Structures in 3-D Culture

**Figure 2:**
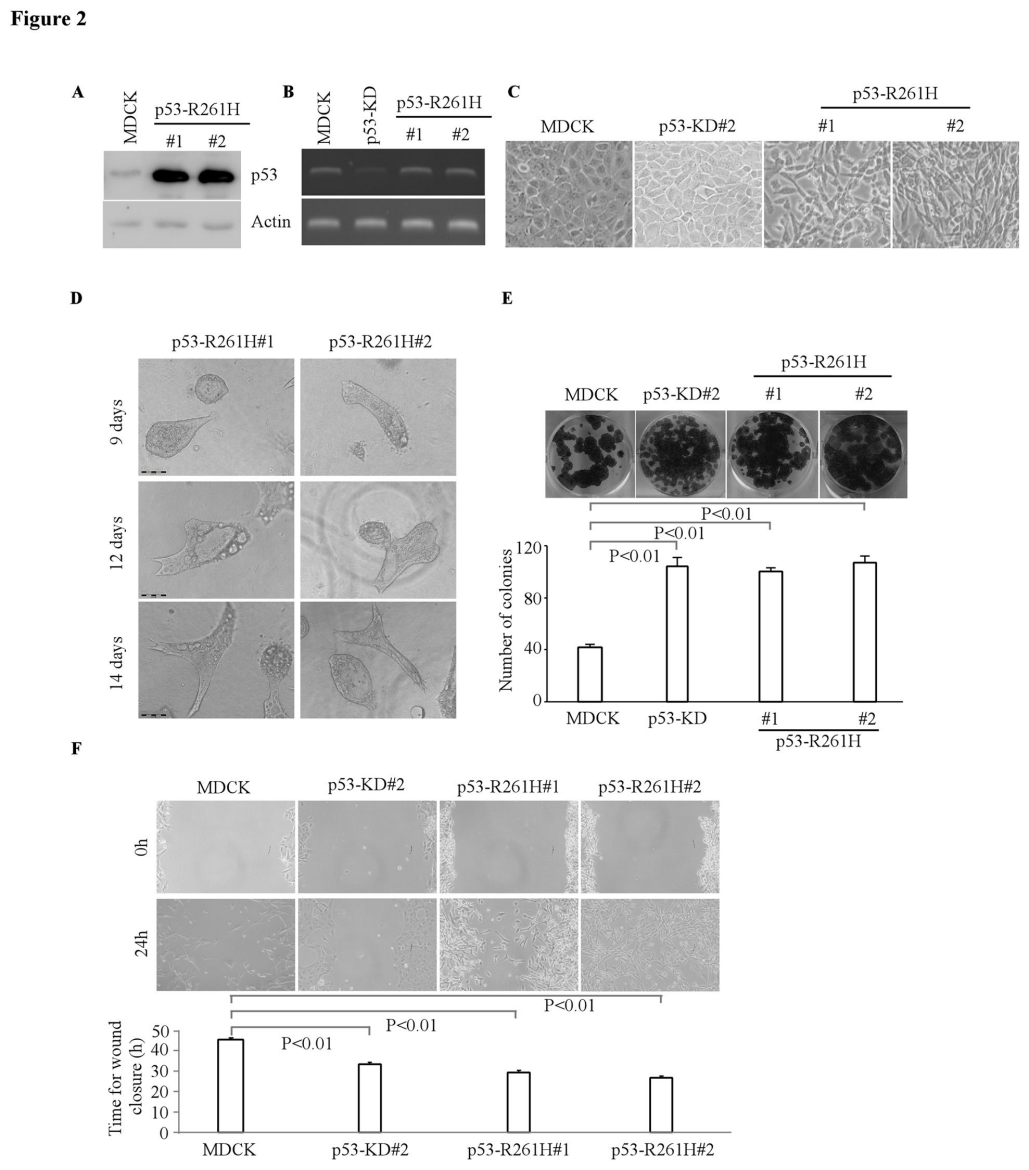
Overexpression of mutant p53-R261H disrupted tubular formation in 3-D culture. **A**, Generation of MDCK cell lines in which siRNA-resistant mutant p53-R261H was stably overexpressed (clones 1 and 2). The protein levels of mutant p53-R261H and actin were measured by Western blotting. **B**, The level of wild-type p53 transcripts was determined by RT-PCR. **C**, Representative images of MDCK cells, MDCK cells with p53 knockdown, or MDCK cells with mutant p53-R261H in 2-D culture (200×). **D**, Representative images of MDCK cells with mutant p53-R261H in 3-D culture. Scale bar: 100 µM. **E**, Top panel: colony formation assay was performed with MDCK cells or MDCK cells with mutant p53-R261H. Bottom panel: the number of colonies was counted and presented as Mean ± SD from three separate experiments. **F**, Wound healing assay was performed with MDCK cells, MDCK cells with p53-KD, or MDCK cells with mutant p53-R261H. Top panel: cell migration was determined by visual assessment of cells migrating into the wound for 24 h using a phase-contrast microscopy. Bottom panel: the time required for wound closure was measured and presented as mean ± SD from three separate experiments.

Next, to determine the effect of contact-site mutant R261H on MDCK cell morphogenesis, we generated multiple MDCK cell lines in which R261H mutant was ectopically expressed (Figure 2A). The mRNA levels of wild-type p53 remain unchanged in MDCK cell lines in which R261H mutant was ectopically expressed (Figure 2B). Compare to the parental MDCK cells (Figure 2C, left panel), we found that MDCK cells with R261H mutant showed remarkable spindle-shaped morphology in 2-D culture (Figure 2C) and grew randomly to form irregular cyst structures in 3-D culture (Figure 2D). In accordance with this, we found that ectopic expression of mutant R261H in MDCK cells significantly enhanced cell growth by colony formation assay (Figure 2E) and cell migration by wound healing assay (Figure 2F). These data indicated that contact-site mutant R261H and conformational mutant R163H shares similar properties in altering cell morphogenesis and in promoting cell growth and migration.

Ectopic expression of mutant p53 R163H or R261H cooperates with knockdown of endogenous wild-type p53 to alter cell polarity

**Figure 3:**
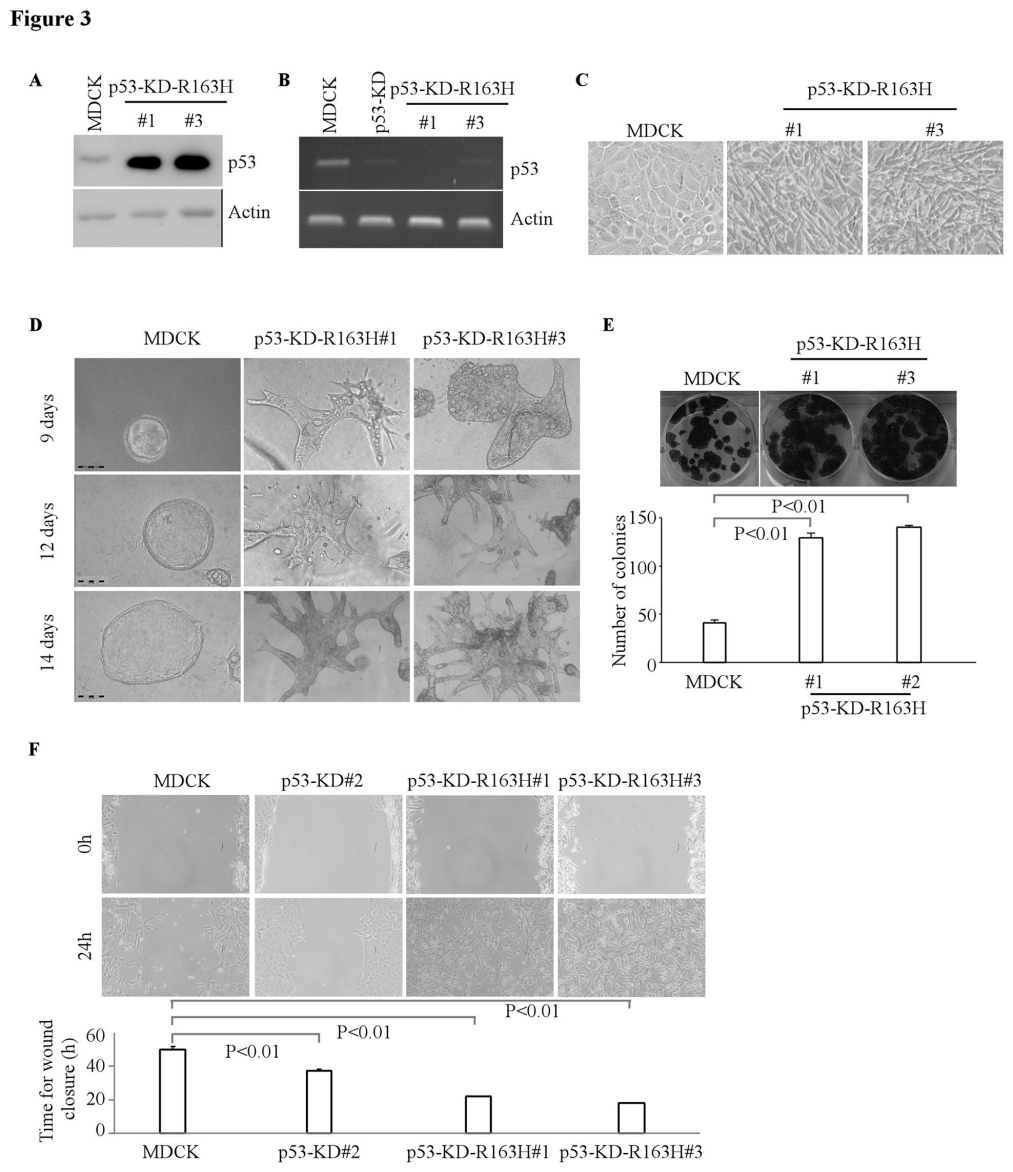
Ectopic expression of mutant p53 R163H cooperates with p53-KD to alter cyst morphology. **A**, Generation of MCF-10A cell lines in which siRNA-resistant mutant p53-R163H was expressed along with knockdown of endogenous wild-type p53. The levels of wide-type p53 and mutant p53-R163H were determined by Western blotting. **B**, The level of wild-type p53 transcripts was determined by RT-PCR. **C**, Representative images of MDCK cells or MDCK cells with p53-KD-R163H in 2-D culture. **D**, Representative images of MDCK cells or MDCK cells with wild-type p53-KD and overexpression of mutant p53-R163H in 3-D culture for 12 d. Scale bar: 100 µM. **E**, Top panel: colony formation assay was performed with MDCK cells or MDCK cells with p53-KD and overexpression of R163H. Bottom panel: the number of colonies was counted and presented as Mean ± SD from three separate experiments. **F**, Wound healing assay was performed with MDCK cells, MDCK cells with p53-KD, or MDCK cells with p53-KD and overexpression of R163H. Top panel: cell migration was determined by visual assessment of cells migrating into the wound for 24 h using a phase-contrast microscopy. Bottom panel: the time required for wound closure was measured and presented as mean ± SD from three separate experiments.

**Figure 4:**
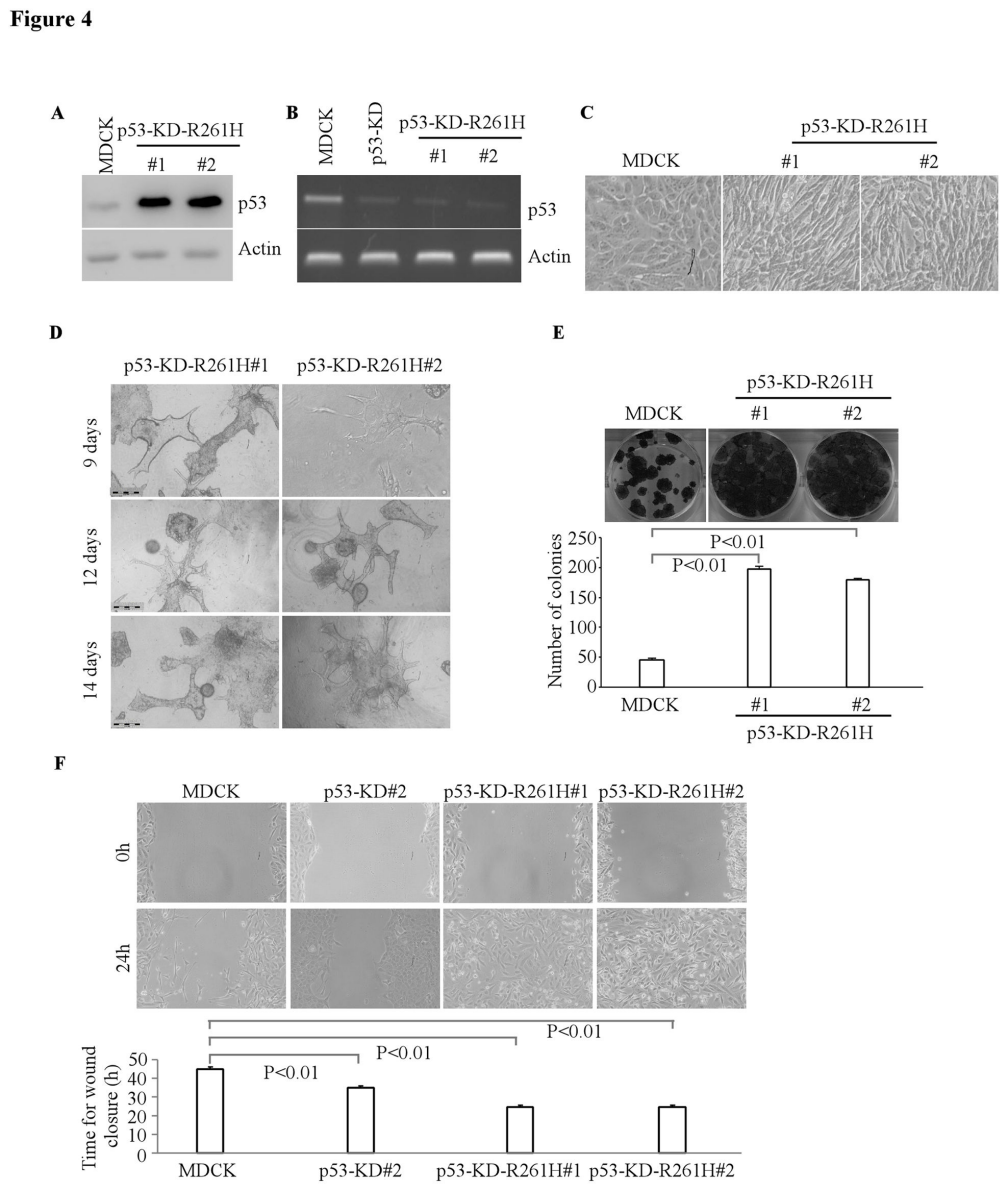
Ectopic expression of mutant p53 R261H cooperates with p53-KD to alter cyst morphology. **A**, Generation of MDCK cell lines in which siRNA-resistant mutant p53 R261H was expressed along with knockdown of endogenous wild-type p53. The levels of wide-type p53 and mutant p53 R261H were determined by Western blotting. **B**, The level of wild-type p53 transcripts was determined by RT-PCR. **C**, Representative images of MDCK cells or MDCK cells with p53-KD-(R261H) in 2-D culture. **D**, Representative images of MDCK cells with p53-KD-R261H in 3-D culture for 12 d. Scale bar: 100 µM. **E**, Top panel: colony formation assay was performed with MDCK cells or MDCK cells with p53-KD-R261H. Bottom panel: the number of colonies was counted and presented as Mean ± SD from three separate experiments. **F**, Wound healing assay was performed with MDCK cells, MDCK cells with p53-KD, or MDCK cells with p53-KD-R261H. Top panel: cell migration was determined by visual assessment of cells migrating into the wound for 24 h using a phase-contrast microscopy. Bottom panel: the time required for wound closure was measured and presented as mean ± SD from three separate experiments.

To determine the effect of mutant R163H or R261H on the morphological alterations in p53-deficient MDCK cells, we generated multiple MDCK cell lines in which R163H or R261H was ectopically expressed individually along with knockdown of endogenous wild-type p53 (Figures 3-4A). Both cell lines expressed high levels of mutant p53 proteins and exhibited low or undetectable levels of endogenous wild-type p53 (Figures 3-4A). The mRNA level of wild-type p53 was efficiently knocked down in MDCK-p53-KD cells, MDCK-R163H-p53-KD cell lines and MDCK-R261H-p53-KD cell lines compared to that in MDCK cells (Figures 3-4B). We found that upon ectopic expression of an individual p53 mutant along with p53-KD, MDCK cells exhibited elongated spindle-shaped phenotype in 2-D culture (Figures 3-4C). In addition, compare to the parental MDCK cells (Figure 3D, left panel), these MDCK cells formed scattered/unordered structures with extensions in 3-D culture (Figures 3-4D). These structures were randomly oriented instead of being perpendicular to the culture plate as in the normal MDCK structures (Figures 3-4D, middle and right panels). Consistently, we found that in p53-KD MDCK cells, ectopic expression of mutant R163H-/R261H further enhanced cell growth and migration (Figures 3-4, E-F). These observations suggest that ectopic expression of mutant R163H or R261H cooperates with knockdown of endogenous wild-type p53 to alter cell polarity in MDCK cells.

### Ectopic expression of mutant R163H or R261H confers MDCK cells to acquire EMT features

**Figure 5:**
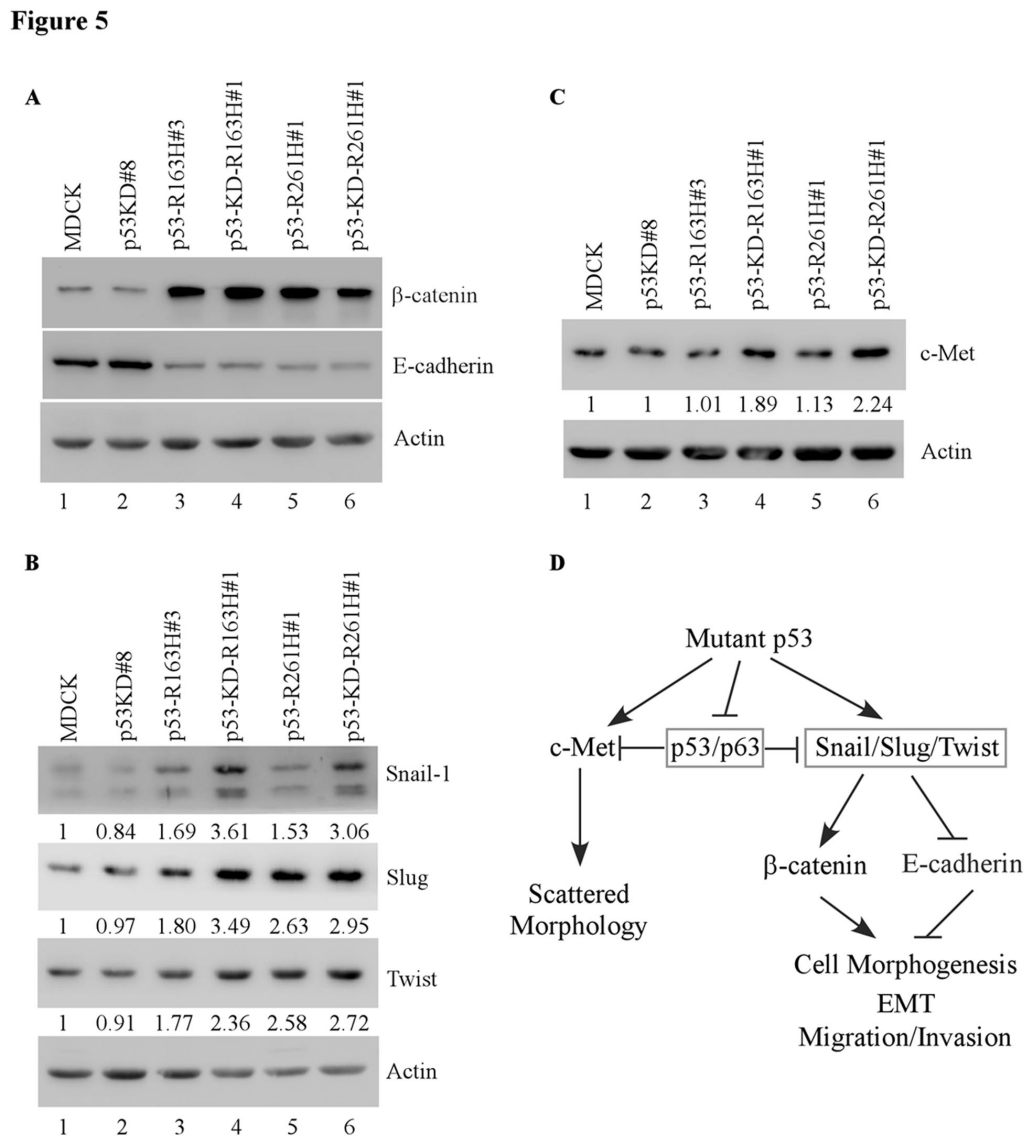
EMT markers are regulated upon ectopic expression of mutant p53, some of which are further enhanced by knockdown of endogenous wild-type p53 in MDCK cells. **A**-**C**, Western blots were prepared with extracts from parental MDCK cells (lane 1), p53-KD MDCK cells (lane 2), MDCK cells in which a mutant p53 was ectopically expressed (lanes 3, 5) and MDCK cells in which a mutant p53 was ectopically expressed along with knockdown of endogenous wild-type p53 (lanes 4, 6). The blots were probed with antibodies against β-catenin (A), E-cadherin (A), Snail (B), Slug (B), Twist (B), c-Met (C) and actin (A-C). The protein levels of EMT markers were quantified and the ratios were labeled under the corresponding bands. **D**, Proposed model of mutant p53 in MDCK cell tubulogenesis.

Loss of normal morphology is the characteristics of EMT [38]. Given that ectopic expression of mutant R163H or R261H leads to alteration of MDCK cell morphology, we sought to determine whether these alterations are due to acquisition of EMT-like properties in MDCK cells. Here, we found that in p53-KD MDCK cells, the levels of EMT markers, such as E-cadherin, β-catenin, Snail-1, Slug and Twist, remained unchanged (Figure 5A-B, compare lane 2 to 1). However, ectopic expression of R163H or R261H increased the level of β-catenin, but decreased the level of E-cadherin, in MDCK cells regardless of knockdown of endogenous wild-type p53 (Figure 5A). In addition, we found that ectopic expression of mutant R163H or R261H alone slightly induced expression of Snail-1, Slug, and Twist in MDCK cells (Figure 5B). Interestingly, the combination of p53-KD with ectopic expression of R163H or R261H markedly enhanced the expression of Snail, Slug, and Twist (Figure 5B, compare lanes 3 and 5 with 4 and 6, respectively). However, mutant p53 was not found to bind to the promoter of the Slug gene by chromatin immunoprecipitation (CHIP) assay (data not shown). The result is consistent with other reports that mutant p53 promotes cell invasion via stabilizing Snail or Slug proteins [27,39]. Finally, we showed that c-MET, the cognate HGF receptor with an oncogenic activity [40], was increased only by the combination of p53-KD and ectopic expression of R163H or R261H (Figure 5C, compare lanes 3 and 5 with 4 and 6, respectively).

## Discussion

Renal cell carcinoma (RCC) is the most common type of kidney cancer and its incidence is increasing [41]. The p53 gene is frequently mutated in RCC and mutations of p53 is associated with prognosis in human RCC [34,35,42]. In addition, mice carrying mutant p53 exhibited defects in terminal renal epithelial differentiation [43]. Thus, it is of clinical significance to determine how mutant p53 controls the morphological differentiation of the renal epithelium. To date, there are no published data on mutant p53 in canine kidney cancer. Therefore, no canine kidney tumor cell lines with mutant p53 are available to test the effect of mutant p53 knockdown. Given that MDCK cell line, which expresses wild-type p53, can recapitulate the *in vivo* epithelial morphogenesis when cultured in a 3-D collagen gel, we developed the 3-D culture model of MDCK cells to study how mutant p53 gain of function is associated with aberrant renal tubulogenesis. Firstly, we found that ectopic expression of mutant p53 R163H or R261H disrupts cyst formation in 3-D culture of MDCK cells. Secondly, we found that mutant p53 R163H or R261H cooperates with p53-KD to disrupt morphogenesis of MDCK cells and form scattered/unordered structures with extensions in 3-D culture. Thirdly, we found that mutant p53 enhances EMT and cooperates with p53-KD to increase the level of c-Met. Taken together, these data indicated that gain of function of mutant p53 alters the normal morphogenesis of MDCK cells via promoting EMT and c-Met expression (Figure 5D).

 It is noteworthy that tight regulation of cyst size, shape and polarization is critical for normal kidney development and functions. Disruption of these regulatory mechanisms leads to an array of diseases including autosomal dominant polycystic kidney disease, stenosis, and cancer [37]. Our previous data [32] and current studies showed that in 3-D culture, p53 knockdown alone is unable to alter MDCK cell morphology, although the cells display enhanced proliferation and migration activities. In present study, we found that ectopic expression of mutant p53 R163H or R261H displays a strong gain of function in altering morphogenesis of MDCK cells in 3-D culture, including disruption of cell polarity and formation of invasive structures with random extensions. These observations are consistent with previous findings that mutant p53 disrupts cell morphogenesis and acini formation coupled with increased cell migration in other experimental system [3,5,44]. Thus, our data imply that these p53 mutants show a gain of function in altering morphogenesis of renal epithelial cells. 

Mutant p53 shows a gain of oncogenic function in driving invasion and metastasis [26,44,45]. In addition, EMT was demonstrated to be a major mechanism responsible for invasiveness and metastasis of cancers. Alterations in adhesion, morphology, cellular architecture and migration capacity are the major events that occur during invasion and metastasis [38]. In this process, transcription factors Snail, Slug and Twist are induced, which in turn repress the expression of E-cadherin [46]. High expression levels of EMT markers, such as N-cadherin and Snail, were found to promote invasiveness in Sarcomatoid RCC [47]. Thus, we sought to determine whether mutant p53 induces EMT in MDCK cells. As expected, we found that mutant p53 R163H or R261H disrupts tubulogenesis of MDCK cells, coupled with decreased expression of E-cadherin and increased expression of β-cateinin. Previously, it has been reported that mutant p53 promote cancer cell invasiveness by stabilizing Snail and Slug proteins [27,39]. Consistently, we also found that mutant p53 upregulates the expression of the transcription factors (Snail, Slug and Twist). These alterations imply that ectopic expression of mutant p53 contributes to the induction of EMT and thus disrupts the tubulogenesis. In addition, it is well known that mutant p53, including conformation mutants (e.g. R175H) and contact mutants (e.g. R273H), are equally capable of binding to p63 to acquire its gain of function through inactivating p63 [48]. P63 is known to inhibit EMT [32,49,50]. Consistently, we found that overexpression of mutant p53 recapitulates the effects of p63 loss [32], suggesting that mutant p53 may disrupt regular cyst formation in 3-D culture partially through counteracting the function of p63 in MDCK cells. 

c-Met, the receptor for HGF, is the product of the *c-met* proto-oncogene and plays a critical role in epithelial-mesenchymal interaction [51]. c-Met regulates cell proliferation and migration, morphogenic differentiation, and organization of 3-D tubular structures during development and tissue repair [52,53]. c-Met receptor is frequently expressed in higher nuclear grade renal cancers, suggesting that deranged expression of c-Met might result in abnormal kidney growth [54]. It has been well-known that HGF, also called scattering factor, and c-MET make MDCK cells scattered in 3-D culture [55]. Previous study showed that mutant p53 enhances c-Met activation in cancer cells, which leads to invasive behavior [56]. Here, we found that expression of c-Met is only increased by ectopic expression of R163H or R261H in MDCK cells with knockdown of endogenous wild-type p53 (Figure 5C), suggesting that mutant p53-enhanced expression of c-MET may be countered by endogenous wild-type p53. Accordingly, we found that knockdown of endogenous wild-type p53 does not, whereas ectopic expression of mutant R163H/R261H partially, promote MDCK cells scattering in 3-D culture. It is likely that induction of c-Met by both p53-KD and ectopic expression of mutant p53 is responsible for formation of scattered structures of MDCK cells. 

In summary, we showed that mutant p53 plays an important role in disrupting tubulogenesis of renal epithelial MDCK cells in 3-D culture and does so through regulating EMT and c-Met. We postulate that ectopic expression of mutant p53 might inhibit p63 activity, or stabilize transcription factors Snail/Slug and Twist, which in turn down-regulates E-cadherin and induces EMT. This process is enhanced by knockdown of endogenous wild-type p53. Moreover, ectopic expression of mutant p53 together with knockdown of endogenous wild-type p53 leads to elevated expression of c-Met, which leads to scattered morphology of MDCK cells in 3-D culture and further promotes cell proliferation and mobility in 2-D culture (Figure 5D).

## Supporting Information

Figure S1 **3-D culture of MDCK cells.**
(TIF)

Figure S2 **Wild-type p53 is not required for tubular formation in MDCK cells.**
(TIF)

Figure S3 **The schematic representation of p53 siRNA targeting sequence and silent mutations in siRNA-resistant mutant p53 cDNA.**
(TIF)
