# Supplementary figures and images for: Mutant p53 Cooperates with Knockdown of Endogenous Wild-Type p53 to Disrupt Tubulogenesis in Madin-Darby Canine Kidney Cells
Source: PLoS One. 2013 Dec 27;8(12):e85624. doi: 10.1371/journal.pone.0085624 (PMC3874028; doi:10.1371/journal.pone.0085624)

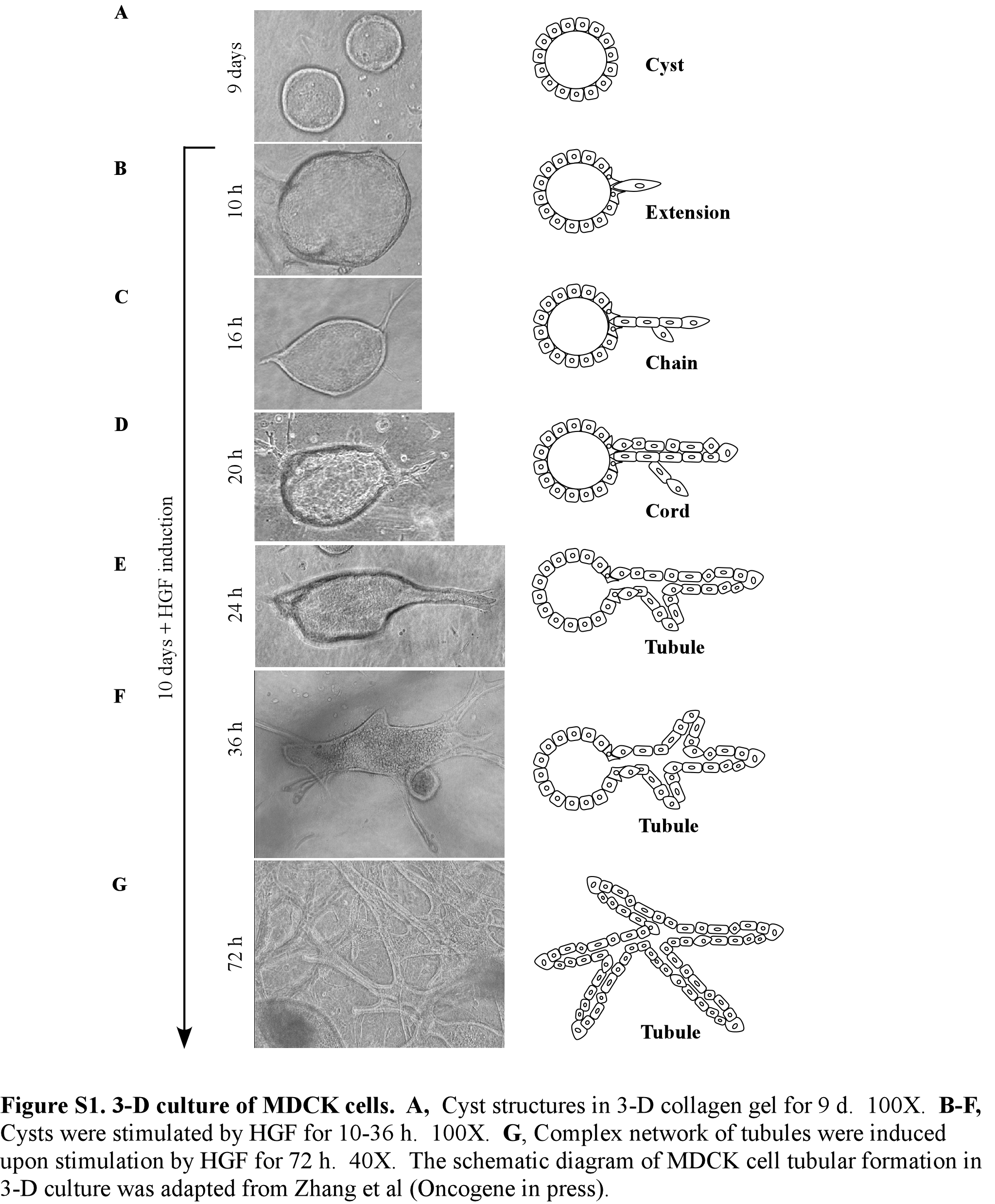

Supplement: Figure S1 — 3-D culture of MDCK cells. (TIF) [file pone.0085624.s001.tif]

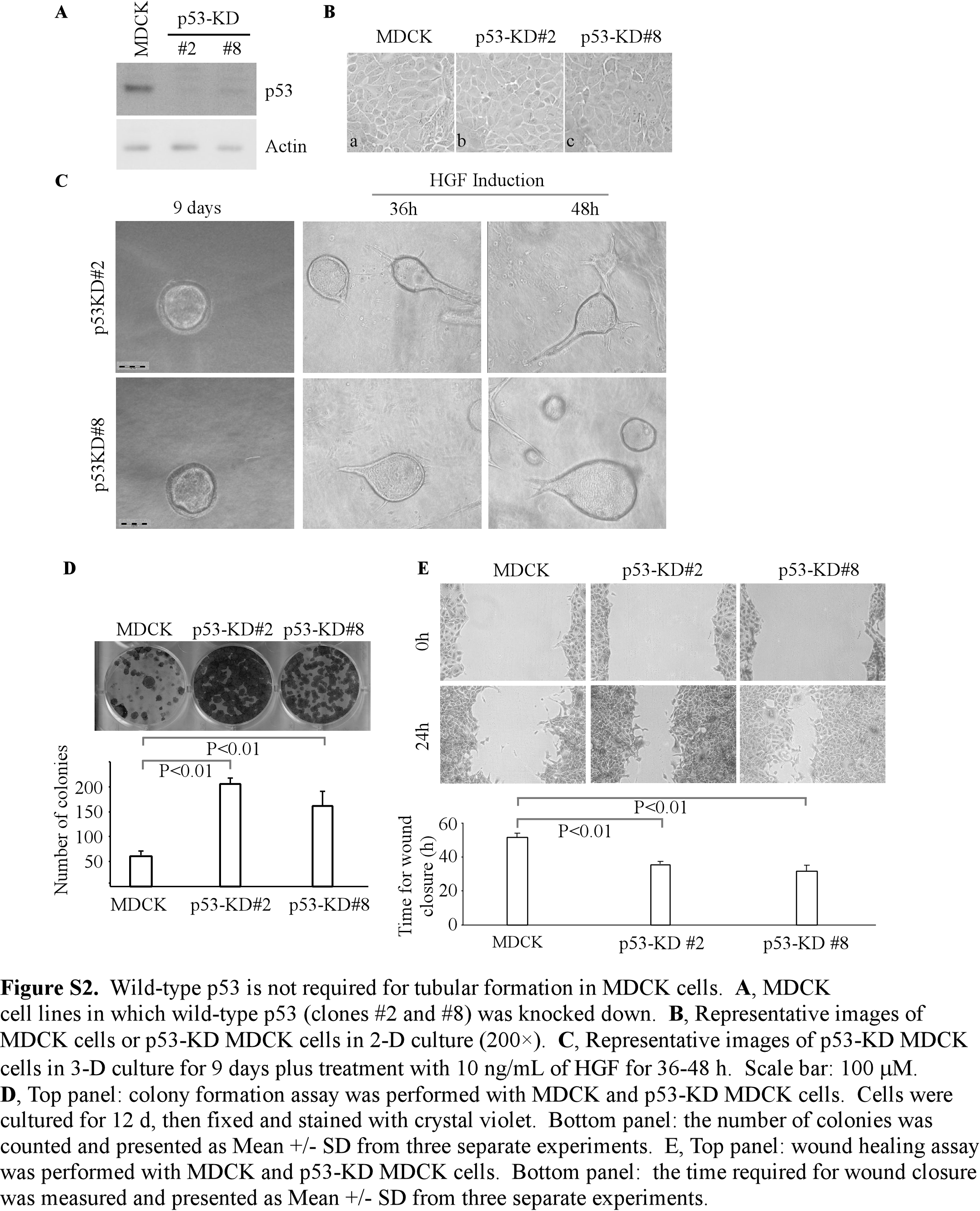

Supplement: Figure S2 — Wild-type p53 is not required for tubular formation in MDCK cells. (TIF) [file pone.0085624.s002.tif]

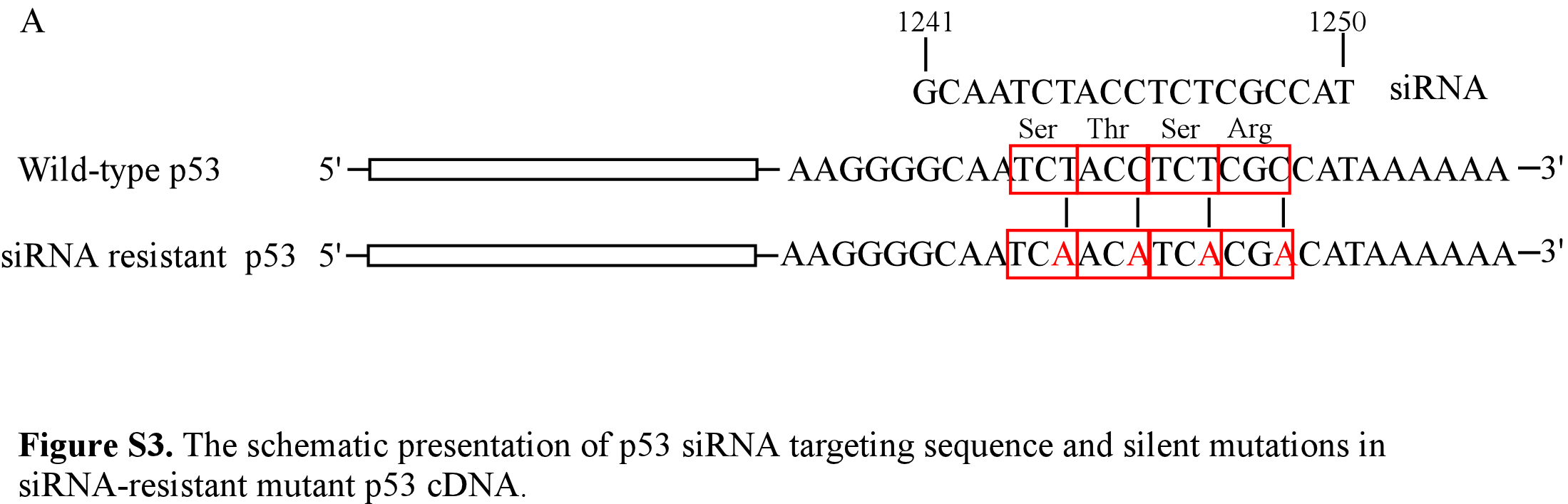

Supplement: Figure S3 — The schematic representation of p53 siRNA targeting sequence and silent mutations in siRNA-resistant mutant p53 cDNA. (TIF) [file pone.0085624.s003.tif]
